# Pyeloplasty for a Child With a Retrocaval Ureter: A Case Report

**DOI:** 10.7759/cureus.36536

**Published:** 2023-03-22

**Authors:** Muhannad Wael, Mohammed A Lubbad, Abdulmalik Jaber, Wael Abuarafeh, Murad Al Hammouri

**Affiliations:** 1 Medicine, An-Najah National University, Nablus, PSE; 2 Urology, Al-Shifa Hospital, Gaza, PSE; 3 Internal Medicine, An-Najah National University, Nablus, PSE; 4 Urology, Saint Joseph Hospital, Jerusalem, PSE

**Keywords:** ureter, double-j stent, surgery, retrocaval, urology

## Abstract

Retrocaval ureter, also known as a circumcaval ureter or pre-ureteral vena cava, is a rare entity in which the patient’s ureter passes backward to the inferior vena cava (IVC). The IVC compresses the upper portion of the ureter resulting in varying degrees of hydroureteronephrosis. We report a case of a child with retrocaval ureter malformation that was incidentally diagnosed while investigating for intermittent right renal colic, which was successfully treated by subcostal pyeloplasty. This case highlights the importance of radiographic imaging to diagnose retrocaval ureter, with computed tomography as a definitive method, but other modalities, including magnetic resonance imaging, ultrasound, and pyelography, are also helpful.

## Introduction

Retrocaval ureter typically arises from anatomical variations of the inferior vena cava (IVC) or its branches and can lead to severe complications if not treated effectively [1]. Hochstetter documented the first known case during an autopsy in 1893 [2]. A retrocaval ureter is a rare condition that occurs more commonly in males (the male-to-female ratio is 3:1 [3]), with mean reported occurrences ranging from 0.07% to 0.13% [3,4]. More than 200 cases have been reported in the literature [5]. We present a case of a retrocaval ureter in a six-year-old female patient.

## Case presentation

A six-year-old girl with tetralogy of Fallot underwent open heart repair surgery when she was seven months old. She was referred to our clinic due to her retrocaval ureter for further evaluation and management. She presented to our clinic with concerns of intermittent dull right flank pain lasting for one year. Ultrasound imaging during the management of a urinary tract infection showed right moderate ureter-hydronephrosis.

**Figure 1:**
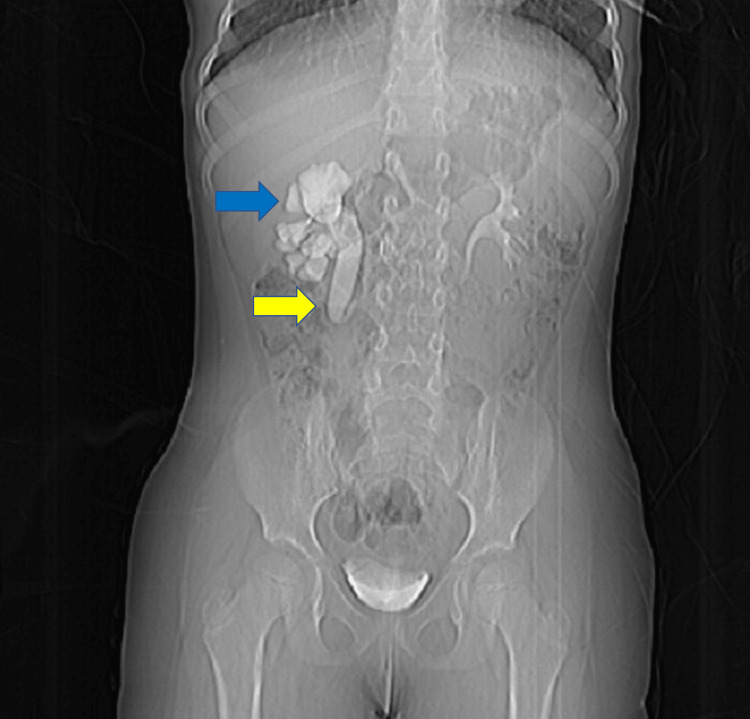
intravenous pyelogram showing the anomaly of the right ureter (blue arrow), which is fishhook-shaped; and moderate ureterohydronephrosis (yellow arrow).

**Figure 2:**
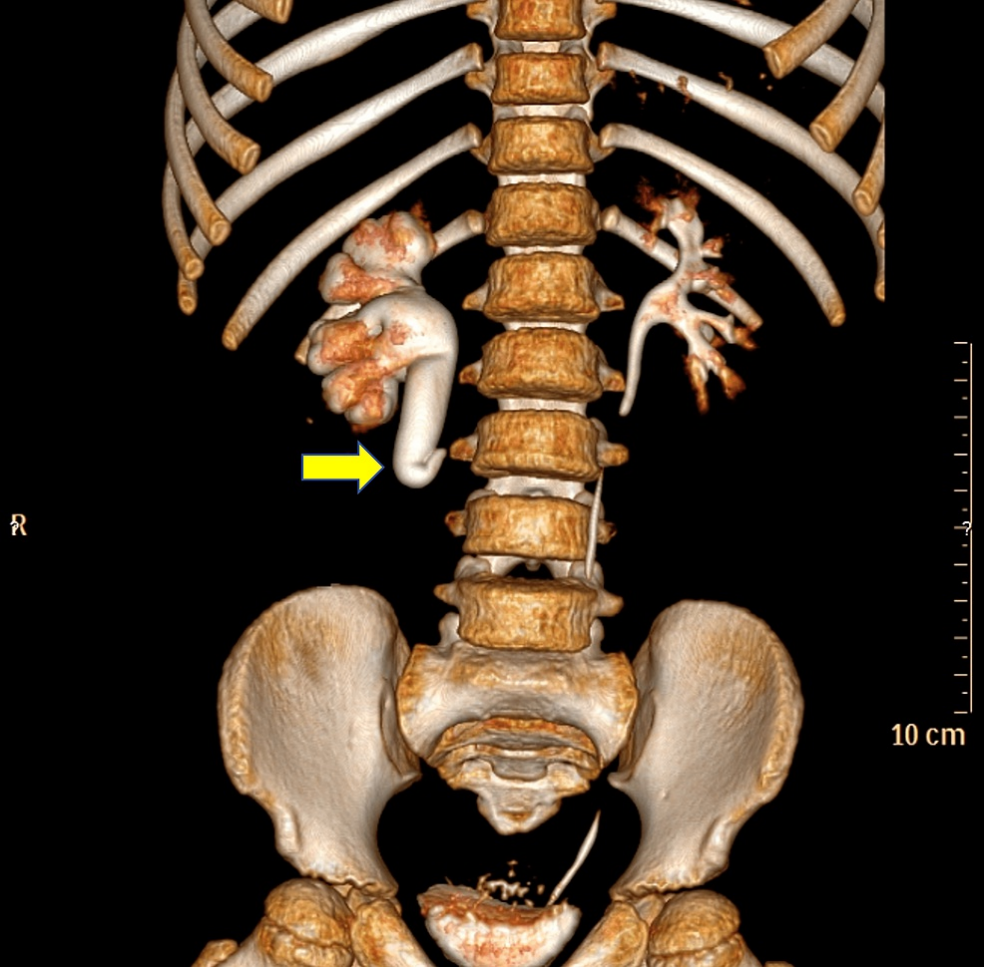
Three-dimensional computed tomography scan showing the right circumcaval ureter anomaly (yellow arrow) that encircles the inferior vena cava.

Her laboratory evaluation results were within reference ranges, including urinalysis, complete blood count, urea, creatinine, and electrolytes (Table 1). A contrast-enhanced computed tomography (CT) scan of her abdomen and pelvis showed right moderate to severe dilation of the upper ureter located behind the inferior vena cava (IVC). A renal mercaptoacetyltriglycine scan revealed a right hydronephrotic, well-functioning, semi-obstructed kidney, with right kidney function at 49% and left kidney function at 51%. The preoperative intravenous pyelogram showed the anomaly of the right ureter, which is fishhook-shaped; and moderate ureterohydronephrosis (Figure 1). A three-dimensional CT scan showed the anomaly of the right ureter (Figure 2).

**Table 1:** Laboratory results on admission.

Urine Analysis	
Color	Yellow
Clarity	Cloudy
pH	6
Specific gravity	1.020
Glucose	120 mg/dl
Ketones	Negative
Nitrites	Negative
Leukocyte esterase	Negative
Blood	3 RBCs
Protein	110 mg/dl
RBCs	1-2 RBCs/hpf
WBCs	3-4 WBCs/hpf
Casts	Negative
Crystals	Occasionally
Bacteria	Negative
Yeast	Negative
Complete Blood Count	
WBC, 10^3^ cells/uL	10
RBC, 10^6^ cells/uL	4.8
HGB, g/dL	12
HCT, %	40
MCH, pg	26
MCHC, g/dL	36
RDW, %	12.5
PLT, 10^3^ cells/uL	250
Kidney Function Test and Electrolytes	
Creatinine, mg/dL	0.40
Ura nitrogen, mg/dL (BUN)	0.47
Chloride (CL)	100 mmol/L
Potassium (K)	4 mmol/L
Sodium (Na)	135 mmol/L

**Figure 3:**
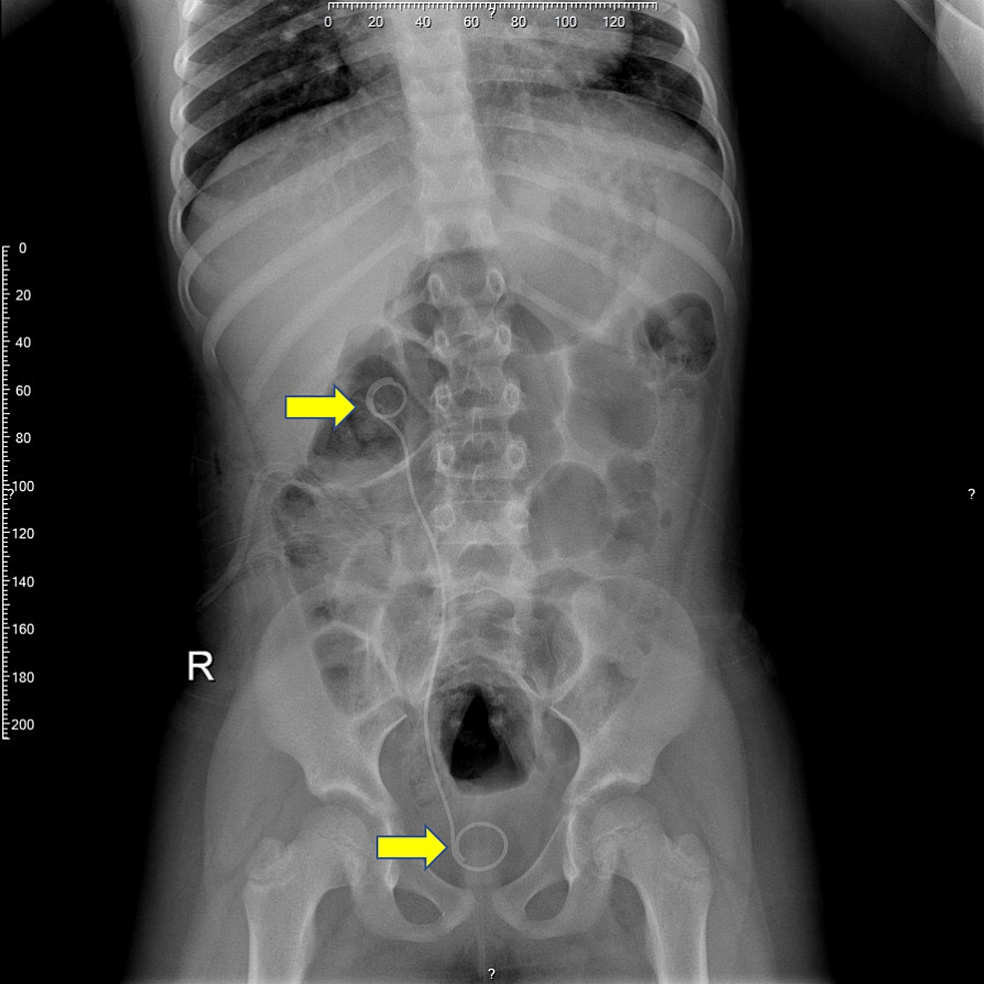
Postoperative x-ray showing the inserted double J stent (yellow arrows).

After discussing the treatment options with the patient's parents, they consented to undergo open surgical correction. The patient received a right lumbotomy approach surgical correction to preserve the kidneys from ureteric obstruction, and she had favorable postoperative evolution. We made a right subcostal incision. The upper segment of the ureter was behind the IVC, and the ureter was transected at the level of lumbar level 2 (L2). Dissection and anastomosis were done for the upper segment to the distal ureter. The postoperative x-ray image is shown in Figure 3.

Five weeks later, we removed the double J-stent. The patient’s follow-up laboratory test results were within reference limits, and her abdominal ultrasound findings were normal. At her six-month follow-up, her abdominal ultrasound was also normal.

## Discussion

Due to the mechanism of developing retrocaval ureter, the right ureter is most likely affected and will run medial and behind the IVC, entrapped between the IVC and the abdominal aorta, and continue in front of and then lateral to the IVC [6]. However, one case of left retrocaval ureter associated with situs inversus and a case of bilateral retrocaval ureter have been reported [7,8]. Symptoms on presentation can vary but typically include flank pain, recurrent unclear urinary tract infections, and obstructive urinary symptoms.

Retrocaval ureter symptoms may present at any age but usually appear in the third and fourth decades of life and are three times more likely to appear in men than women [9]. Therefore, our case involving a six-year-old female child was surprising. The condition is associated with many congenital anomalies related to the urinary system or other systems and can include horseshoe kidneys [10], polycystic kidney disease [11], congenital solitary kidney, the ectopic position of the kidneys, hypospadias, situs inversus, and congenital heart diseases (our patient has tetralogy of Fallot) [12].

There are two main types of retrocaval ureter based on the site of ureter entrapment visible on radiographs. Type 1 (a fish-hook deformity, or S-shape) is more common and involves the ureter crossing the IVC at the level of the L3 vertebra (low loop), producing the typical appearance of a fish hook deformity on pyelography. This type mainly causes marked hydronephrosis due to obstruction caused by ureter entrapment. Type 2 retrocaval ureter (a sickle-shape deformity) is less common than type 1 and involves the ureter crossing the IVC at a higher level (mainly at the level of the renal pelvis; the high loop), producing the typical appearance of sickle-shape deformity on radiographs. However, the hydronephrosis associated with this type is minimal and does not usually require surgical intervention [5].

While many imaging modalities can be used to diagnose retrocaval ureter, CT of the abdomen-pelvis is currently the best method and can exclude other abnormalities involving the urinary system. Magnetic resonance imaging (MRI) is not superior to CT, but it can be used in cases of kidney failure when CT contrast media are contraindicated [13]. Ultrasound imaging is a good way to assess the extent of hydroureter nephrosis.

The best management for asymptomatic retrocaval ureter patients with normal kidney tissue scans is watchful waiting, and patients with documented marked kidney damage should undergo a nephrectomy for the affected kidney. On the other hand, symptomatic patients should be managed surgically. The open surgery approach is used in most cases; however, the laparoscopic approach offers several benefits over the open method, including shorter hospitalization, better outcomes, and fewer complications.

Whether it is better to preserve or cut the retrocaval segment of the ureter is a subject of debate. A report of six retrocaval ureter cases describes the successful treatment with pyeloplasty at the level of the kidney pelvis, with preservation of the retrocaval segment of the ureter [14]. However, other authors suggested that the decision to preserve or resect depends on the intraoperative and imaging findings, concluding that the retrocaval segment should be preserved if this segment is healthy, of normal width, and not kinked. Otherwise, the resection of the retrocaval segment should be considered [15].

## Conclusions

The retrocaval ureter is a rare congenital anomaly that, if symptomatic, usually presents in male patients in the third or fourth decades of life. We described a case of a retrocaval ureter in a six-year-old female child that was managed surgically with successful outcomes. CT imaging remains the preferred method to detect the retrocaval ureter, but other modalities such as MRI, ultrasound, and pyelography are viable options.
